# Encapsulation and Melanization Are Not Correlated to Successful Immune Defense Against Parasitoid Wasps in *Drosophila melanogaster*

**DOI:** 10.3390/cells14010046

**Published:** 2025-01-03

**Authors:** Lilla B. Magyar, István Andó, Gyöngyi Cinege

**Affiliations:** Innate Immunity Group, Institute of Genetics, HUN-REN Biological Research Centre, 6726 Szeged, Hungaryando@brc.hu (I.A.)

**Keywords:** *Drosophila melanogaster*, encapsulation, melanization, hemocyte, parasitoid wasp, eclosion, immune response

## Abstract

Parasitoid elimination in *Drosophila melanogaster* involves special hemocytes, called lamellocytes, which encapsulate the eggs or larvae of the parasitoid wasps. The capsules are melanized, and metabolites of the melanization reaction may play a potential role in parasitoid killing. We have observed a variation in the melanization capacity of different, commonly used *D. melanogaster* strains, such as Canton-S, Oregon-R, and BL5905, BL6326. In this work, we aimed to clarify a possible connection between the effectiveness of capsule melanization and the success of parasitoid elimination following infection with *Leptopilina* parasitoid wasps. Circulating hemocytes and lamellocyte attachment were visualized by confocal and epifluorescence microscopy using indirect immunofluorescence. Expression profiles of the *PPO2* and *PPO3* prophenoloxidase genes, which encode key enzymes in the melanization reaction, were detected by qRT-PCR. Parasitization assays were used to analyze fly and wasp eclosion success. Active encapsulation and melanization reactions against *Leptopilina boulardi* were observed in the BL5905 and the BL6326 strains, though restricted to the dead supernumerary parasitoids, while fly and wasp eclosion rates were essentially the same in the four examined *D. melanogaster* strains. We conclude that encapsulation and melanization carried out by *D. melanogaster* following *L. boulardi* infection have no impact on survival.

## 1. Introduction

Insects possess sophisticated cellular and humoral immune defense reactions to protect the organisms from pathogens [1,2,3]. The cellular immune response of *Drosophila melanogaster* is mediated by the hemocytes and involves phagocytosis of microbes by plasmatocytes and encapsulation of large foreign particles, such as eggs and larvae of parasitoid wasps, by the combined action of plasmatocytes and lamellocytes [4]. Lamellocytes are large, flattened cells that differentiate following infection by parasitoid wasps or after cuticle wounding and constitute the main encapsulating hemocyte type of *D. melanogaster* [5].

The encapsulation is often associated with a melanization reaction, which involves prophenoloxidases (PPOs) released into the hemolymph by the crystal cells and activated by a cascade of serine proteases [6], resulting in the deposition of dark melanin, thus hardening the capsule [7]. Melanization was long considered an important process in the immune response of insects [8] because, besides its involvement in the capsule formation around parasitoids, it also hardens the clot of cuticle wounds [9,10,11] caused by different mechanical traumas, such as the insertion of the parasitoid wasps’ ovipositor. Melanization is a multistep chain reaction consisting of the oxidation of tyrosine and the generation of quinones, which polymerize to form melanin [12]. Meanwhile, toxic products, reactive oxygen species, are formed [7]. The generation of melanized capsules was considered to be a general feature of *D. melanogaster* larvae to inhibit the development of various parasitoids, hence aiding the fruit fly in overcoming parasitization and undergoing complete development [5,7,13,14,15]. It was shown that prophenoloxidase 2 (PPO2), produced by the crystal cells, along with prophenoloxidase 3 (PPO3), specific for lamellocytes, are involved in the melanization of the capsules [16,17]. It has long been hypothesized that the death of the parasitoids in *D. melanogaster* is the result of encapsulation and the melanization reaction, which generates reactive oxygen species and attracts toxic compounds to the vicinity of the parasitoid [18]. However, the actual key factors involved in parasite killing have not been clearly identified.

*Leptopilina boulardi* and *Leptopilina heterotoma* are two frequently studied parasitoid wasp species that generally develop successfully in *D. melanogaster*. This has been attributed to immune-suppressive factors present in their venom, which are introduced into the host larvae by the female wasp during oviposition [19,20,21]. The immune-suppressive molecules may inhibit the proliferation, differentiation, and adhesion of the host hemocytes, hence preventing the formation of multicellular capsules [21]. Some parasitoid wasp species lay more than one egg in the *Drosophila* host. In these cases, parasitoids employ different strategies to eliminate the wasp overload. It was shown that supernumerary wasps were regularly killed by one dominant parasitoid [22,23,24]. The thus killed wasps represent a large amount of foreign material for the host to eliminate, while the dominant wasp completes development.

We found that the efficiency of encapsulation and melanization of *L. boulardi* is strikingly different between the Canton-S and Oregon-R wild-type *D. melanogaster* strains, and the BL5905 and BL6326 isogenic W[1118] strains with genetically engineered backgrounds, which carry a mutation in the white gene. This observation raised the question of how these strains defend themselves and whether there is a difference in the wasp and fly eclosion rates among them. To elucidate this, the four *D. melanogaster* strains were examined following infection with *L. boulardi* and *L. heterotoma* parasitoid wasps, with regard to hemocyte composition, encapsulation and melanization efficiency, *PPO2* and *PPO3* gene expression, and fly and wasp eclosion rates.

Our results highlighted that encapsulation and melanization are not general phenomena in parasitized *D. melanogaster* and that these reactions do not affect the eclosion success of the flies. Rather, they are primarily involved in isolating and clearing supernumerary parasitoids killed by the suppression programs of the dominant wasp.

## 2. Materials and Methods

### 2.1. Insect Stocks and Culturing

*D. melanogaster* wild-type Oregon-R and Canton-S strains, and BL5905 and BL6326 stocks with w[1118] genotype were from Bloomington Drosophila Stock Center. The *L. boulardi* (G486) and *L. heterotoma* (14) parasitoids were kindly supplied by Prof. Todd Schlenke (University of Arizona, Tucson, AZ, USA) and were kept on *D. melanogaster* Oregon-R. Each strain was maintained at 25 °C on standard yeast–cornmeal food.

### 2.2. Generation of Wasp-Induced Samples

Sixty-second instar *D. melanogaster* larvae were incubated with 15 female *L. boulardi* or *L. heterotoma* parasitoid wasps at 25 °C for 6 h. Among these conditions, 100% of the host larvae were infected. We selected the infected larvae based on the melanized spot of the oviposition on the cuticle.

### 2.3. Parasitoid Monitoring and Analysis of Capsule Melanization

Forty-eight or seventy-two hours post-infection, *D. melanogaster* larvae were dissected individually under a stereomicroscope in 30 µL volume of Schneider’s medium (Lonza, Basel, Switzerland) complemented with 5% fetal bovine serum (GIBCO) and 0.01% 1-phenyl-2-thiourea (Sigma, Kanagawa, Japan) (CSM) to block melanization after dissection, on multispot microscope slides (Hendley-Essex, Loughton, UK). Individual larvae were examined for the developing parasitoids, their viability, and signs of melanization. Live parasitoids were motile. Three independent experiments were performed, each with 60 *D. melanogaster* larvae.

### 2.4. Indirect Immunofluorescence and Image Analysis

The *L. boulardi* parasitoids were isolated 72 h post-infection in CSM, fixed for 10 min with 2% paraformaldehyde, washed three times in PBS for 5 min each, blocked and permeabilized with 0.1% BSA in PBS complemented with 0.1% Triton X-100. Samples were incubated with the lamellocyte-specific L1 (anti-Atilla), a panhemocyte-specific (anti-Hemese) monoclonal antibody [25], and the T2/48 negative control [26], each in the form of undiluted hybridoma supernatants. After 3 washes with PBS for 5 min, the samples were incubated with anti-mouse Alexa Fluor 488 goat secondary antibody (Invitrogen, Carlsbad, CA, USA, 1:1000 dilution) and DAPI for 45 min. Samples were washed again as above and covered with Fluoromount G medium and coverslip. Analysis was performed with an epifluorescence microscope (Zeiss Axioscope (Jena, Germany) 2 MOT, (for hemocytes) or an Olympus (Tokyo, Japan) FV1000 confocal LSM microscope (for parasitoids). Lamellocytes were counted with the ImageJ program (version 1.54i) using nuclear DAPI and cellular L1 fluorescence. Three independent experiments were performed with 20 larvae in each.

### 2.5. Quantitative RT-PCR

Thirty age-matched *L. boulardi* infected (72 h post-infection) and naïve *D. melanogaster* larvae were used to isolate RNA with an RNeasy mini kit (Qiagen, Hilden, Germany) based on the manufacturer’s instruction. Twenty-five microliter cDNA was generated from 1 µg RNA using the RevertAid First Strand cDNA Synthesis Kit (Thermo Scientific, Waltham, MA USA) and the Oligo(dT)18 Primer. For quantification, 2 µL of 10 times diluted cDNA was applied with PerfeCTa SYBER Green SuperMix (Quanta bio, Beverly, MA, USA) in a Rotor-Gene Q (Qiagen) qPCR platform. The following oligonucleotide primer sets were used: PPO2 specific forward 5′-CGAGGCCATTCACCAAGGAT-3′ and reverse 5′-GGTTGGGCGACAGAATGGAT-3′; PPO3 specific forward 5′-ACCAGTTGAGGGTTGGCATC-3′ and reverse 5′-CTTAGCGTCATCCAGCACGA-3′; *rp49* (FBgn0002626), housekeeping ribosomal protein-encoding gene-specific forward 5′-ACAGGCCCAAGATCGTGAAG-3′ and reverse 5′-ACGCACTCTGTTGTCGATAC-3′. The following reaction conditions were used: 95 °C 2 min, 45 cycles at 95 °C for 10 s, 57 °C for 45 s and 72 °C for 15 s. Two independent experiments were conducted with technical duplicates. For data analysis, Rotor-Gene Q Series Software and Q-Rex 1.0 were used. To interpret gene expression levels, the ∆∆Ct method was used. The *rp49* housekeeping gene was used for the normalization of cycle threshold (ct) values.

### 2.6. Parasitization Assay

Seventy early second instar *D. melanogaster* larvae were exposed to 17 female *L. boulardi* parasitoid wasps at 25 °C for 6 h. These conditions usually resulted in 100% infection of the host larvae. To test the parasitization success, 48 h after the infection, 10 larvae were dissected and analyzed. When each larva carried at least one parasitoid, the respective vial was considered available for the assay. Four to six independent parasitization assays were carried out with each fly line. The eclosed flies and wasps were counted. Infected flies and the uninfected controls were kept under similar conditions.

### 2.7. Statistical Analysis

Statistical analysis was carried out with the RStudio v.2024.04 software. Normal distribution of data was assessed by the Shapiro–Wilk test. For the statistical analysis of the hemocyte count and lamellocyte percentage data, Kruskal–Wallis test and pairwise Wilcoxon tests with multiple testing corrections (Benjamini–Hochberg p.adjust.method) were performed, while gene expression and eclosion data were assessed by one-way ANOVA followed by Tukey HSD test. All data were expressed as mean ± standard error of the mean. A *p*-value less than 0.05 was considered significant, where * = *p* ≤ 0.05, ** = *p* ≤ 0.01, and *** = *p* ≤ 0.001.

## 3. Results

### 3.1. Lamellocytes Differentiate in the Examined D. melanogaster Strains

To clarify the role of encapsulation and capsule melanization in parasitoid killing by *D. melanogaster*, we used four *Drosophila* strains: Oregon-R, Canton-S, BL5905, and BL6326, and two parasitoid wasp species: *L. boulardi* and *L. heterotoma*.

**Figure 1:**
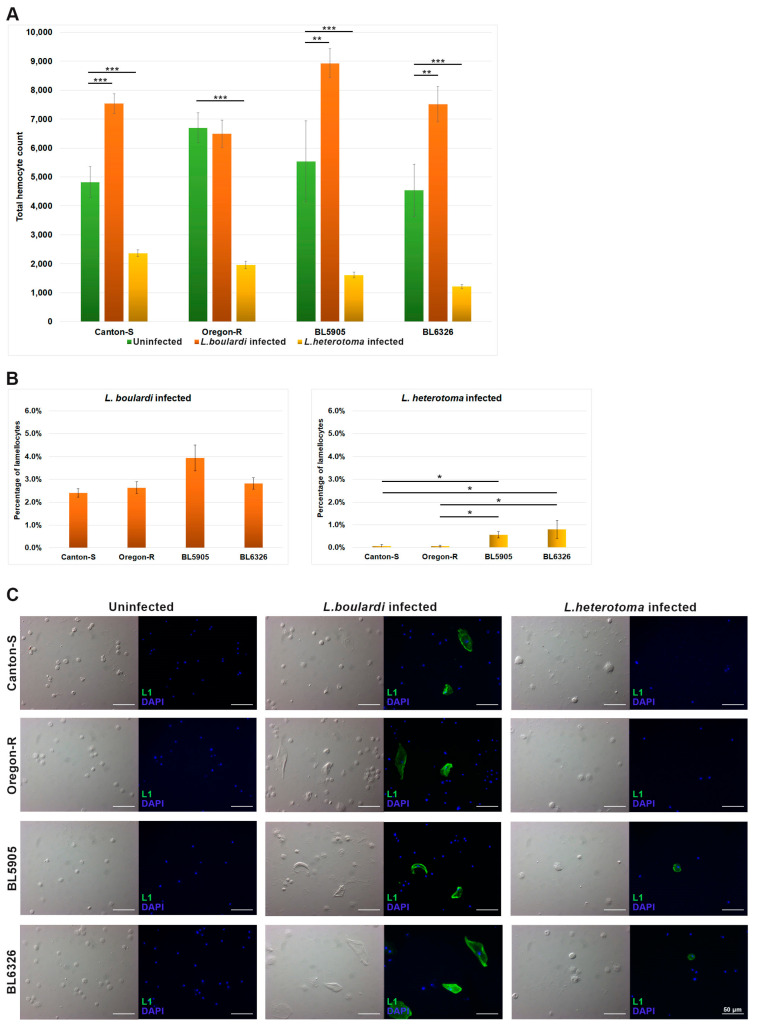
Hemocyte composition in *D. melanogaster* larvae following parasitoid wasp infection. Hemocyte isolates of wasp-infected (72 h post-infection) and age-matched naïve larvae were examined. The total hemocyte number (**A**) and the ratio of the lamellocytes related to the total hemocyte count (**B**) were analyzed in four *D. melanogaster* strains. Three independent experiments were performed with 20 larvae each. The error bars indicate the standard error of the mean. Kruskal–Wallis test and pairwise Wilcoxon tests were used for statistical analysis. *p*-values: <0.05 = *; <0.01 = **; <0.001 = ***. (**C**) Indirect immunofluorescence analysis of hemocytes using the anti-L1 antibody to detect lamellocytes and DAPI to visualize the nuclei. Images were generated with an epifluorescence Zeiss Axioscope 2 MOT microscope. Representative images are shown.

First, to obtain insights into the cellular immune responses of the analyzed *D. melanogaster* strains, hemocytes of *L. boulardi*- and *L. heterotoma*-infected fruit fly larvae were isolated 72 h after infection, and their circulating hemocyte compositions were compared to that of age-matched naïve samples. *L. boulardi* infection caused a significant increase in the total hemocyte count of each strain (*p*-values: 0.00077–0.0061), except in Oregon-R (*p*-value: 0.38). However, after the *L. heterotoma* attack, a dramatic decrease was detected when compared to that of the naïve animals (*p*-values: 6.1 × 10^−12^–1.8 × 10^−5^) (Figure 1A).

Next, we analyzed lamellocyte differentiation, the main hemocyte type responsible for the encapsulation reaction. To obtain the percentage of the lamellocytes, their number was related to the total hemocyte counts. In the uninfected larvae, lamellocytes were not present, in the *L. boulardi*-infected ones, these cells represented 2.4–3.9% of the circulating hemocytes (Figure 1B). However, following *L. heterotoma* infection, not only did the total hemocyte number decrease dramatically (Figure 1A), but the proportion of lamellocytes among the hemocytes was also strongly reduced, representing only 0.06–0.8% of the total hemocyte count (Figure 1B). In *L. heterotoma*-infected larvae, the lamellocyte number was lower in the Canton-S and the Oregon-R than in the BL5905 and BL6326 strains (Figure 1B,C), and hemocyte lysis was strongly reflected in the morphology of L1-positive cells, which were smaller and more rounded when compared to the large flattened lamellocytes that differentiated after *L. boulardi* infection (Figure 1C). The samples incubated with the T2/48 control antibody [23] were negative.

### 3.2. Encapsulation and Melanization Efficiency Varies Across D. melanogaster Strains

Lamellocytes produce PPO3, a key enzyme responsible for the melanization of the capsule formed around the parasitoids infecting *D. melanogaster* [17]. Although lamellocytes differentiated following *L. boulardi* infection and were normally present in the circulation of each examined strain (Figure 1B,C), we found variations in the melanization frequency. Specifically, 72 h after infection with *L. boulardi*, 97% of the BL5905 and 90% of the BL6326 larvae, while only 15% of the Canton-S and 10% of the Oregon-R larvae carried at least one melanized parasitoid. Hence, we aimed to study and compare the parasitization efficiency and the encapsulation and melanization reactions in these *D. melanogaster* strains.

**Figure 2:**
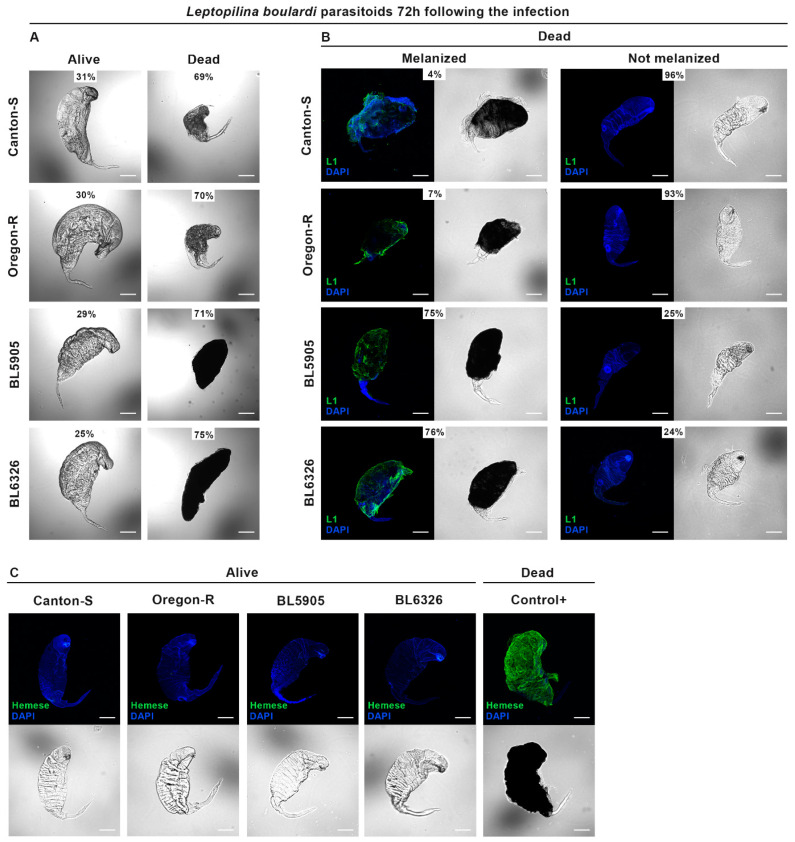
Survival of *L. boulardi* 72 h post-infection does not correlate with encapsulation and melanization efficiency. Three independent experiments were completed, each with 60 *D. melanogaster* larvae. Images were taken with an Olympus FV1000 confocal LSM microscope. The scale bars represent 100 μm. (**A**) Most of the host larvae carried one live and more dead *L. boulardi* parasitoids. The live/dead ratio of parasitoids is indicated for each strain. Representative images are shown. (**B**) Indirect immunofluorescence analysis of dead parasitoids using the L1 lamellocyte-specific monoclonal antibody and DAPI to visualize the nuclei. The ratio of melanized and not melanized dead parasitoids is indicated. (**C**) Indirect immunofluorescence analysis of live *L. boulardi* parasitoids using the anti-Hemese monoclonal antibody and DAPI to visualize the nuclei. As positive control, supernumerary dead *L. boulardi* parasitoids, isolated from BL5905, were used.

*L. boulardi*-infected larvae were individually dissected, and the isolated parasitoids were counted and checked for viability, capsule formation, and melanization. The wasps laid 2–12 eggs into each *D. melanogaster* larva, and at 48 h post-infection, all parasitoids were in larval stage and showed highly similar features as those monitored at 72 h post-infection (see below, Table 1 and Figure 2A). Seventy-two hours after infection, depending on the *D. melanogaster* strain, we detected a single live parasitoid in 87–97% of the hosts (Table 1). Besides the live, dominant parasitoid, in general, 1–11 dead (supernumerary) wasp larvae were present. Only 3–7% of the hosts carried two live parasitoids (Table 1). Live parasitoids could not be observed in 7% of the BL5905 and in 8% of the BL6326 hosts.

The dead parasitoids were smaller than the live dominant individuals, and the live/dead ratio was similar in each strain (Figure 2A). Melanization of the dead parasitoids was often observed in BL5905 and BL6326, while it was a very rare event in the Canton-S and the Oregon-R strains (Figure 2B).

To analyze lamellocyte adhesion to the dead parasitoids, the dead wasp larvae were collected and analyzed with the lamellocyte-specific L1 monoclonal antibody by indirect immunofluorescence. Only a few parasitoids, 4% in the Canton-S and 7% in the Oregon-R strains, carried lamellocytes, which were always associated with the melanization reaction (Figure 2B), while in the BL5905 and BL6326 strains, most of the dead parasitoids (75% and 76%) were encapsulated by lamellocytes and all these capsules were melanized (Figure 2B). To analyze hemocyte attachment to the live parasitoids, the panhemocyte-specific anti-Hemese monoclonal antibody was used in indirect immunofluorescence analysis. We found that live *L. boulardi* parasitoids were free from hemocytes (Figure 2C).

Contrary to *L. boulardi*, *L. heterotoma* deposited only one egg into 72–94% of the hosts, the rest (6–28%) carried two or sometimes three parasitoids, among which one wasp was always alive. Before pupariation, each host larva carried at least one live parasitoid. In 60% of the hosts with two or three parasitoids, the supernumerary wasps were dead. 

### 3.3. Expression of the PPO2 and PPO3 Genes

The *PPO2* and the *PPO3* genes are known to be involved in capsule melanization [17]. We analyzed the expression of these genes in each examined *Drosophila* strain by qRT-PCR on samples isolated from *L. boulardi*- and *L. heterotoma*-infected larvae 72 h post-infection and those of age-matched naïve animals.

**Figure 3:**
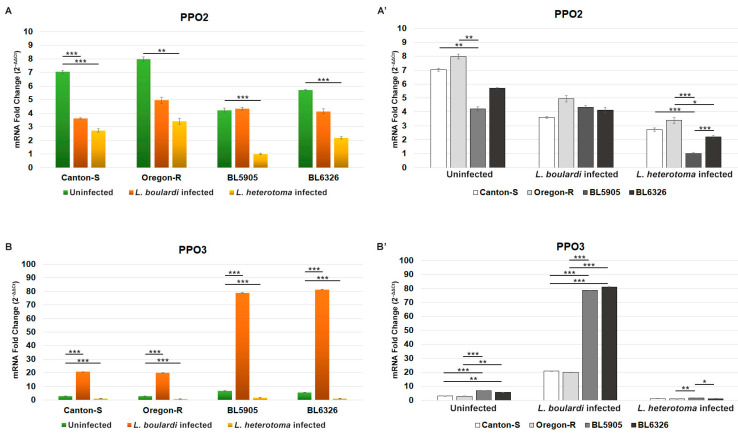
Expression of *PPO2* (**A**,**A’**) and *PPO3* (**B**,**B’**) prophenoloxidase genes following infection with *L. boulardi* and *L. heterotoma* parasitoid wasps. The same data are shown in (**A**,**A’**) and (**B**,**B’**), in different interpretations. The error bars indicate standard error of the mean of four data points. Two independent experiments were carried out, with two technical replicates in each. ANOVA and Tukey HSD were used for statistical analysis. Significant differences are labeled. * = *p* ≤ 0.05; ** *p* ≤ 0.01; *** = *p* ≤ 0.001. ΔΔCt was calculated by normalizing ΔCt against the lowest values, the *L. heterotoma*-infected Oregon-R samples in (**A**,**A’**), and the *L. heterotoma*-infected BL5905 in (**B**,**B’**).

We found that expression of the *PPO2* gene decreased in the wasp-infected samples when compared to the naïve specimens (*p*-values: 0.000001–0.0057) except in the *L. boulardi*-infected BL5905 (Figure 3A). Interestingly, expression of the *PPO2* gene showed significant variations in the naïve (*p*-values: 0.0057, 0.00103) and the *L. heterotoma*-infected (*p*-values: 0.0000059–0.033) samples, depending on the examined strains (Figure 3A’).

Following infection with *L. boulardi*, expression of the *PPO3* increased significantly in all *D. melanogaster* strains when compared to that of naïve larvae (*p*-values: 0–0.0000016), and this difference was more evident in the BL5905 and BL6326 (Figure 3B,B’). Infection by *L. heterotoma* caused a significant decrease in the *PPO3* expression in each strain (*p*-values: 10^−7^–0.0007). As housekeeping control, the ribosomal protein-encoding *rp49* (FBgn0002626) gene was used. In negative controls, templates were generated without the addition of reverse transcriptase.

### 3.4. Parasitoid Emergence Does Not Correlate with the Efficiency of Encapsulation and Melanization

**Figure 4:**
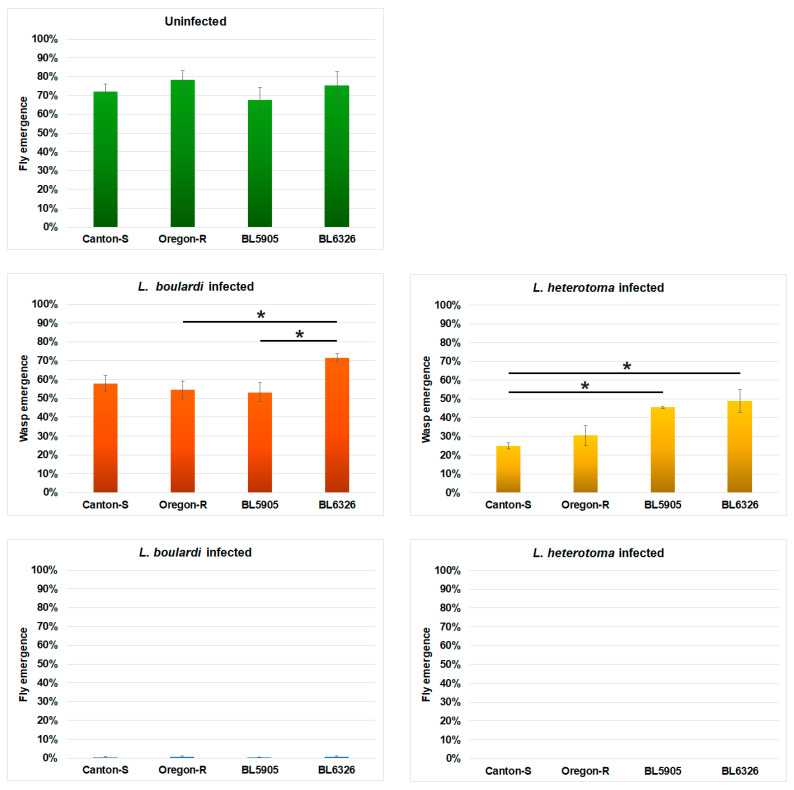
Eclosion success following infection with *L. boulardi* and *L. heterotoma*. Four to six independent experiments were carried out with 60 *D. melanogaster* larvae in each. The error bars indicate the standard error of the mean. ANOVA and Tukey HSD were used for statistical analysis. *p*-values: <0.05 = *.

Larvae of the BL5905 and the BL6326 strains encapsulated *L. boulardi* parasitoids and melanized capsules at a higher rate and showed increased *PPO3* gene expression when compared to Canton-S and the Oregon-R (Figure 2 and Figure 3A); hence, we aimed to study how these processes influenced the fly and wasp emergence rates. Following *L. boulardi* infection, fly eclosion rates were very low (0.21–0.63%); however, wasp eclosion rates were 53–71% in each strain (Figure 4). The highest wasp eclosion rate was observed in the BL6326 strain. These results showed that eclosion rates did not correlate with the parasitoid encapsulation and melanization efficiency (Pearson correlation coefficient = 0.47).

Moreover, following infection with *L. heterotoma*, no flies emerged from any of the *D. melanogaster* strains, and wasp eclosion rates were 25–49%, and hence lower than those of *L. boulardi*. In BL5905 and BL6326, more *L. heterotoma* wasps emerged than in the Canton-S and the Oregon-R strains, which was a significant difference between the Canton-S and the W[1118] strains (*p*-values: 0.016–0.034).

## 4. Discussion

Encapsulation-associated melanization is a well-known reaction of insects as part of their cellular immune response against larger pathogens [2,11]. In *D. melanogaster,* capsule-melanization is preceded by the differentiation of special hemocytes, the lamellocytes, which encapsulate parasitoid eggs or larvae [5,27,28]. Later, the capsule is melanized by the activity of phenoloxidases, PPO2, produced in the crystal cells, and PPO3, produced by lamellocytes [17]. The physical isolation of the parasitoid by the melanin layer and the toxic by-products of the melanization reaction are believed to be involved in parasite killing, hence helping the host to overcome parasitization and undergo complete development [7,18]. Encapsulation and melanization are not general phenomena in insects for parasite killing. For example, in *Drosophila paramelanica*, hemocyte-mediated encapsulation was not detected [7,14], and several species, such as *Drosophila ananassae* or *Zaprionus indianus,* exhibit successful anti-parasitoid responses without melanotic encapsulation [29,30,31].

In this work, we aimed to elucidate the relationship between parasitoid wasp encapsulation, capsule melanization efficiency, and eclosion success of flies and wasps in the most frequently used insect model organism, *D. melanogaster*. We used four *D. melanogaster* strains that are often used in the laboratories: Canton-S, Oregon-R, BL5905, and BL6326. As *L. boulardi* is the most extensively studied parasitoid wasp infecting *D. melanogaster* larvae, this species was first used in the analysis. We found that *L. boulardi* G486 females laid 2–12 eggs in each larva of the examined *D. melanogaster* strains. Superparasitism, when a single host is injected with more parasitoid eggs, is not a rare phenomenon, but in solitary parasitoids, only one wasp survives to adulthood [24]. The number of parasitoids within one host also depends on the wasp–host incubation time [32]. Superparasitism is an energetically costly process, which requires varied, complex physiological suppression programs from the wasp and still not entirely elucidated mechanisms to control and allow the development of only one dominant wasp neonate. It is known that within one *D. melanogaster* host, the supernumerary *Ganaspis xanthopoda* endoparasitoids develop to the first larval instar, where their development is physically trapped by a multicellular envelope that was formed during late embryogenesis [24]. Moreover, supernumerary parasitoid larvae may be eliminated by direct physical attack, physiological suppression, selective starvation, or accidental injury [23]. Furthermore, in the case of *L. heterotoma*, as well as *Asobara tabida,* intraspecific killing by biting was described [23].

At 72 h post-infection, we found that in each strain, most of the hosts carried 1 live and 1–11 dead *L. boulardi* parasitoids (Table 1, Figure 2A). The presence of only one live *L. boulardi* parasitoid in most of the hosts (Table 1) suggested that the killing of the supernumerary wasps was likely due to the natural wasp competition. We found that the anti-parasitoid reactions of the Oregon-R and the Canton-S strains were remarkably different from those detected in the BL5905 and the BL6326 strains. In the Canton-S and the Oregon-R strains, *L. boulardi* encapsulation and capsule melanization rarely occurred, and in the BL5905 and BL6326, it happened frequently (Figure 2B). Nonetheless, the ratio of the dead/live wasps was similar in all four strains (Figure 2A). Hence, we anticipate that encapsulation and melanization contributed to the isolation of the already dead parasitoids and did not affect the development of the dominant wasp. This hypothesis is also supported by the wasp emergence rates from the infected strains (Figure 4), which did not correlate with the encapsulation and melanization ability of the hosts (Figure 2B). Moreover, BL6326, the strain presenting the most intense encapsulation and melanization activity, had the highest *L. boulardi* wasp emergence (Figure 2B and Figure 4).

Encapsulation of the dead supernumerary wasps gave the impression that the host was actively killing these parasitoids; however, only 7% of the BL5905 and 8% of the BL6326 third instar larvae carried exclusively dead *L. boulardi* parasitoids (Table 1), and there were no such hosts in the Canton-S and the Oregon-R strains. Moreover, the fly eclosion rates were very low (0.21–0.63%) in each *D. melanogaster* strain, and the wasp eclosion rates were not higher in Canton-S and Oregon-R, the strains with the lowest encapsulation and melanization rates (Figure 4), as would have been expected. Hence, we hypothesize that the majority of the BL5905 and BL6326 larvae, carrying only dead parasitoids, were not able to survive and complete development. Furthermore, because in the Canton-S and the Oregon-R strains, at least one live parasitoid was present in each larva, and fly eclosion rates were low (0.21–0.63%), but not zero, killing of a few dominant wasps may occur during pupal stages, hence allowing a few flies to emerge. Very poor survival of the *L. boulardi*-infected *D. melanogaster* host was also reported previously [33,34,35,36,37], and fly eclosion rate increased when facultative bacterial endosymbionts as *Spiroplasma* or *Wolbachia* that are vertically transmitted from mother to offspring were present in the host [33,38]. These bacteria compete with the parasitoid wasps for host lipids, hence conferring endosymbiont-mediated protection [38]. Successful development of *L. boulardi* in *Drosophila* can be related to immune-suppressive factors introduced in host larvae by the wasp during oviposition [19,20,21]. Studies with *Ceratitis cosyra* parasitized by *Diachasmimorpha longicaudata* or *Psyttalia cosyrae* wasp species also demonstrated that encapsulation efficiency does not correlate with the eclosion success [39], indicating the importance of other defense mechanisms.

While *L. boulardi* applies superparasitism, *L. heterotoma* regularly lays only one egg in the host [40,41]. The venom of *L. heterotoma* causes rapid lysis and blocks differentiation of hemocytes in *D. melanogaster* [42]; hence, it is not surprising that the total hemocyte count decreased significantly, the number of L1 positive cells was strongly reduced (Figure 1A,B), and the absence of encapsulation and melanization reactions was obvious. Interestingly, *L. heterotoma* wasp emergence rates were not higher, despite the host possessing a reduced hemocyte number, when compared to that of *L. boulardi* (Figure 4), which in general caused an increase in the total hemocyte count (Figure 1A,B).

Infection with *L. boulardi* induces cellular and humoral anti-parasitoid immune reactions of the fruit fly larva, which interplay to eventually kill the parasite [1,3,43,44]. While cellular responses include the proliferation of hemocytes and differentiation of lamellocytes, which encapsulate parasitoids, humoral immunity is mediated mostly by the fat body of the larva. However, the anti-parasitoid humoral immune mechanisms of *D. melanogaster* are not well characterized because only a few secreted immune effector molecules were shown to be involved in this process, such as thioester-containing proteins [45], a C-type lectin called lectin-24A [46], and serine proteases with key roles in the melanization reaction and activation of the Toll pathway [47,48]. The *PPO3* gene is expressed by the lamellocytes [17,49,50,51,52], cells which differentiate following *L. boulardi* parasitoid wasp infection in each analyzed strain; hence, the significant upregulation of this gene was evident (Figure 3B). Moreover, in the BL5905 and the BL6326 strains, induction of the *PPO3* gene was remarkably higher than that observed in the Canton-S and the Oregon-R strains (Figure 3B), and we hypothesize that the elevated number of lamellocytes attached to the parasitoids in BL5905 and BL6326 (Figure 2B) could further activate expression of the *PPO3* gene. The significant decrease in the *PPO3* gene expression following *L. heterotoma* infection (Figure 2B) can be the result of the low hemocyte load (Figure 1B) caused by the rapid venom-mediated lysis [53,54]. The *PPO2* gene is expressed by the crystal cells, and we detected a decreased expression in each wasp-infected sample, except in the *L. boulardi*-infected BL5905 sample, where it did not change when compared to the naive ones (Figure 3A). The decrease in *PPO2* gene expression might be explained by the rupture of the crystal cells, which occurred after injury caused by the ovipositors of parasitoid wasps [6].

Melanization has already been proven to be an important factor in insect immunity, as it is involved in the survival of flies from infection by Gram-positive bacteria and fungi [16]. In this work, we could not exclude that encapsulation by lamellocytes and capsule melanization in *D. melanogaster* may contribute to the killing of other parasitoid species [17,41]. As *D. melanogaster* shows high sensitivity to *L. boulardi* and *L. heterotoma*, no protective effects of these factors could be detected. However, in some insects and several *Drosophila* species, effective, mostly humoral factors such as genotoxins and pore-forming toxins, encoded by *cytolethal distending toxin B* and *hemolysin E*-like genes, captured by horizontal gene transfer strongly enhance the success of the host in fighting parasitoids [31,55]. Studying these humoral factors could lead to the discovery of new immune modules instrumental in effective anti-parasitoid reactions.

## Figures and Tables

**Table 1:** Percentage of the *D. melanogaster* larvae carrying one, two, or no live *L. boulardi* parasitoids. The majority of the hosts carried one dominant live and more supernumerary, dead parasitoids. Detection was conducted 72 h following *L. boulardi* infection. The values represent mean of three independent experiments with 60 larvae each.

*D. melanogaster* Strain	No Wasps Alive	One Wasp Alive	Two Wasps Alive
Canton-S	0%	93%	7%
Oregon-R	0%	97%	3%
BL5905	7%	88%	5%
BL6326	8%	87%	5%

## Data Availability

The data presented in this study are available from the corresponding author upon request.
